# Usefulness of a novel method for the screening of deep vein thrombosis by using a combined D-dimer- and age-based index before total hip arthroplasty

**DOI:** 10.1371/journal.pone.0172849

**Published:** 2017-02-24

**Authors:** Norio Imai, Dai Miyasaka, Hayato Shimada, Ken Suda, Tomoyuki Ito, Naoto Endo

**Affiliations:** 1 Division of Comprehensive Care of Geriatrics, Niigata University Graduate School of Medical and Dental Science, Niigata, Japan; 2 Division of Orthopedic Surgery, Department of Regenerative and Transplant Medicine, Niigata University Graduate School of Medical and Dental Science, Niigata, Japan; 3 Department of Orthopaedic Surgery, Saiseikai Niigata Daini Hospital, Niigata, Japan; Medical University Innsbruck, AUSTRIA

## Abstract

Plasma D-dimer level is clinically useful for diagnosing patients with suspected deep vein thrombosis (DVT). However, the cut-off value for the D-dimer level remains controversial and undetermined with regard to total hip arthroplasty (THA). The objective of this study was to estimate the efficacy of an age- and D-dimer-based index for diagnosing DVTs in asymptomatic cases before THA. We enrolled 224 patients with no symptoms associated with DVT before THA. All the patients underwent ultrasonography, and the plasma D-dimer level was recorded about 1 month preoperatively. The optimal cut-off value was calculated using multiple logistic regression and receiver operating curve analyses. DVTs were detected in 13 patients (5.8%) using ultrasonography. Multiple logistic regression analysis demonstrated that age (odds ratio [OR]: 1.13; *p* = 0.007) and D-dimer value (OR: 1.74; *p* = 0.003) were related to the existence of preoperative DVT. A DVT index (0.12 × age + 0.45 × the D-dimer value) of 8.15 was the most reasonable cut-off value according to the receiver operating curve analysis. This value caused 100% sensitivity and 70.1% specificity, with an area under the curve (AUC) of 0.905 (range, 0.836–0.975). For age and D-dimer value, the AUCs were 0.828 (0.749–0.907) and 0.716 (0.522–0.910), respectively. This study demonstrated that age and D-dimer index can be useful in screening patients for DVTs before THA. This DVT index is also easy to calculate and may be clinically significant.

## Introduction

Pulmonary thromboembolism (PTE) following deep vein thrombosis (DVT) is one of the general complications that can be related to significant mortality after orthopedic operation, particularly total hip arthroplasty (THA) [1–4]. Therefore, in addition to appropriate triage, earlier and more reliable diagnoses of PTE and DVT are necessary for the affected patients to improve their outcomes [5–8]. Søresen et al. [9] described that the relative risks for patients with DVT vary from 1.60 for myocardial infarction to 2.19 for stroke in the first year after the thrombotic event and are also increased during the subsequent 20 years of follow-up. Søgaard et al. [10] argued that patients with DVT or PTE are at increased risk of dying, particularly within the first year after diagnosis, and during the entire 30 years of follow-up, with DVT or PE as an important cause of death. Therefore, we believe that DVT is not only a postoperative complication after THA, but also an important factor that influences prognosis. Although many studies on the occurrence of DVT postoperatively have been conducted [1–8], few reports on the presence of DVT preoperatively are available. A number of physicians believe that almost all patients, before undergoing THA, are not complicated by DVT; however, screening of DVT before THA may be clinically valuable.

If a patient who is undergoing THA has a DVT, physicians should consider anticoagulation therapies, such as warfarization, inferior vena cava filter placement, and thrombolytic therapy, or the procedure may need to be postponed. Thus, the presence of preoperative DVT should not be ignored, but its clinical symptoms are unreliable. Venography and ultrasonography are considered the most suitable methods for DVT diagnosis despite their several limitations. Venography is invasive, expensive, and not easily repeatable, whereas ultrasonography requires experienced technicians, equipment, and labor force [8]. Since these procedures are time consuming and/or costly, repeated assessments of DVT are almost impossible at institutions where many orthopedic operations are conducted annually. Given this reason, we routinely perform ultrasonography before and after THA in our institutions to screen DVT.

Therefore, plasma markers are commonly used to diagnose DVTs. However, debate exists about the level of plasma D-dimer, a fibrin degradation product of the crosslinked fibrin mesh. Nevertheless, D-dimer level is considered a useful diagnostic marker for patients with suspected DVT [11,12].

Schouten et al. [13] argued that the application of age-adjusted cut-off values to the D-dimer value (0.1 × age [years] × true D-dimer value [μg/mL]) considerably elevates specificity without altering sensitivity in patients aged >50 years with suspected PTE. Therefore, the cut-off value of the D-dimer is still controversial and undetermined. The purpose of this study was to evaluate the relation of the existence of DVT before THA and the use of plasma D-dimer value, age, sex, body height, body weight, and body mass index for the screening of DVTs.

## Materials and methods

This retrospective study was approved by the ethics committee of our institution (Niigata University Medical and Dental Hospital, Niigata, Japan), and written informed consent was obtained from all the participants. Two hundred and thirty-eight patients underwent primary THA at Niigata University Medical and Dental Hospital from January 1, 2010 to December 31, 2014. Patients with a previous history of thromboembolism and those who received anticoagulation or antiplatelet agents for pre-existing cardiac or cerebrovascular diseases were excluded. Subsequently, 224 patients were enrolled. The preoperative diagnosis was osteoarthritis of the hip in 198 patients and osteonecrosis of the femoral head in 26.

The age, sex, body height, body weight, body mass index (weight in kilograms divided by the square of the height in meters), and plasma D-dimer level of the patients were recorded about 1 month preoperatively. All the patients underwent ultrasonography around the same time that the plasma D-dimer level was measured. Plasma D-dimer levels were collected for routine health purposes for this study. Skilled radiologists who were blinded to the D-dimer value performed all the examinations. Ultrasonography was conducted with compression and color Doppler imaging in B-mode for the common femoral, superficial, popliteal, and calf veins bilaterally. DVT was diagnosed by a loss of compressibility of the vein, the existence of intraluminal echogenicity, and the absence of venous flow. Plasma D-dimer levels were measured using latex agglutination turbidimetry via the CA8000 (Sysmex Co., Kobe, Japan). The normal range is <0.5 μg/mL.

Fisher's exact test was performed to compare the qualitative data such as the number of males or females and the presence or absence of DVT. Unpaired Student’s t-test was conducted to analyze the quantitative data such as age, body height, body weight, body mass index, and D-dimer value. We used multiple logistic regression analysis to determine which of the following factors contributed to the presence of DVT: age, sex, body height, body weight, body mass index, and D-dimer value. Receiver operating characteristic (ROC) curves were also applied to determine the cut-off value with regard to the statistically significant factor(s). The area under the curve (AUC) was also calculated from ROC curves, and the cut-off value was determined by the Youden index [14,15]. SPSS (version 21; SPSS Inc., Chicago, IL, USA) was used for all statistical analyses, and a *p* value <0.05 was considered statistically significant.

## Results

None of the 224 patients showed symptoms suggestive of DVT such as calf pain, tenderness, and swelling. Of these patients, 13 (5.8%) had DVTs with distal type detected by ultrasonography. The patients’ clinical features are presented in Table 1. Patients with and without DVT demonstrated that age (*p* < 0.001) and D-dimer value (*p* = 0.048) were significantly different (Table 1).

**Table 1:** Patients’ characteristics.

	Total patients (n = 224)	Patients with DVT (n = 13)	Patients without DVT (n = 211)	*p* value^✝^
Age (years)	58.8 ± 10.6	69.1 ± 5.3	58.2 ± 10.5	<0.001
Sex (male/female)	46/178	1/12	45/166	0.476*
Side (right/left)	117/107	5/8	112/99	0.395*
Body height (cm)	155.5 ± 9.2	155.6 ± 9.2	153.0 ± 9.0	0.397
Body weight (kg)	56.9 ± 12.3	52.7 ± 8.2	57.1 ± 12.5	0.138
Body mass index (kg/m^2^)	23.6 ± 4.2	22.7 ± 3.7	23.6 ± 4.2	0.432
Preoperative D-dimer (μg/mL)	1.49 ± 1.33	2.86 ± 2.93	1.39 ± 1.20	0.048
White blood cell count(/μL)	5947 ± 1446	6117 ± 1219	5858 ± 1580	0.674
Hemoglobin (g/dL)	13.0 ± 1.4	12.6 ± 1.4	13.0 ± 1.6	0.308
Hematocrit (%)	40.3 ± 3.9	39.7 ± 3.9	40.8 ± 4.0	0.511
Platelet count (×10^4^/μL)	23.7 ± 6.8	22.3 ± 4.6	23.8 ± 6.9	0.479
APTT (s)	30.7 ± 4.0	29.9 ± 3.2	30.7 ± 4.0	0.518
PT (%)	106.3 ± 15.0	102.7 ± 8.5	106.4 ± 15.5	0.452
PT–INR	0.98 ± 0.07	0.99 ± 0.04	0.98 ± 0.08	0.758

Data are presented as mean ± standard deviation.

DVT, deep vein thrombosis; APTT, activated partial thromboplastin time; PT, prothrombin time; PT–INR, prothrombin time–international normalized ratio.

*, Fisher’s exact test.

^✝^, comparison between patients with DVT and without DVT.

According to multiple logistic regression analysis, when age, sex, body height, body weight, body mass index, and D-dimer value were independent variables, age and D-dimer value were associated with preoperative DVT (Table 2). Multiple logistic regression analysis indicated that age and D-dimer value were considered significant risk factors (coefficient regressions = 0.12 and 0.45, respectively; Table 3).

**Table 2:** Multiple logistic regression analysis for preoperative deep vein thrombosis.

Independent variable	Odds ratio	95% CI	*p* value
Age (year)	1.13	1.034–1.238	0.007
Sex (female)	5.51	0.262–11.559	0.272
Side (right)	0.90	0.902–1.231	0.507
Bogy height (cm)	1.06	0.867–1.303	0.345
Body weight (kg)	0.95	0.743–1.201	0.642
Body mass index (kg/m^2^)	1.15	0.635–2.062	0.653
D-dimer (μg/mL)	1.74	1.209–2.504	0.003

CI, confidence interval.

**Table 3:** Multiple logistic regression analysis for age and D-dimer value.

Independent variable	Coefficient regression	Odds ratio	95% CI	*p* value
Age (years)	0.12	1.12	1.046–1.208	0.002
D-dimer (μg/mL)	0.45	1.56	1.172–2.081	0.002

CI, confidence interval.

**Fig 1:**
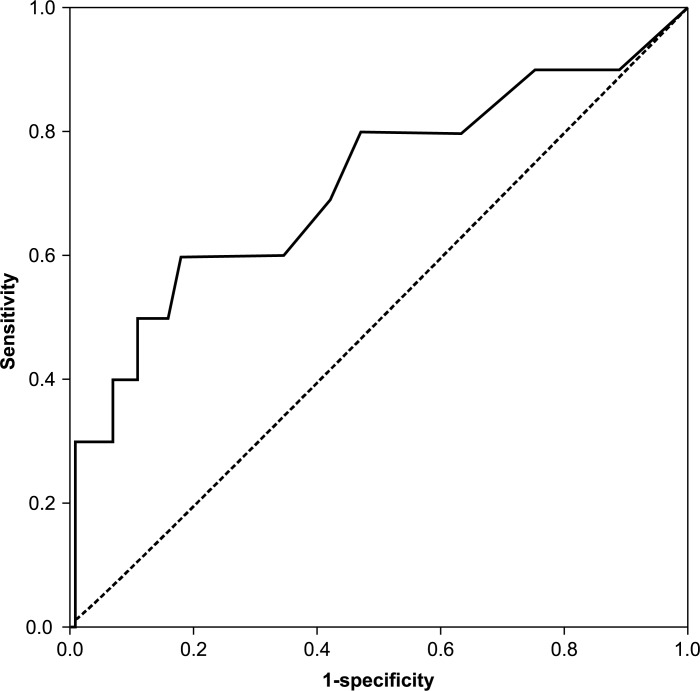
Receiver operating characteristic curve of the D-dimer value preoperatively.

**Fig 2:**
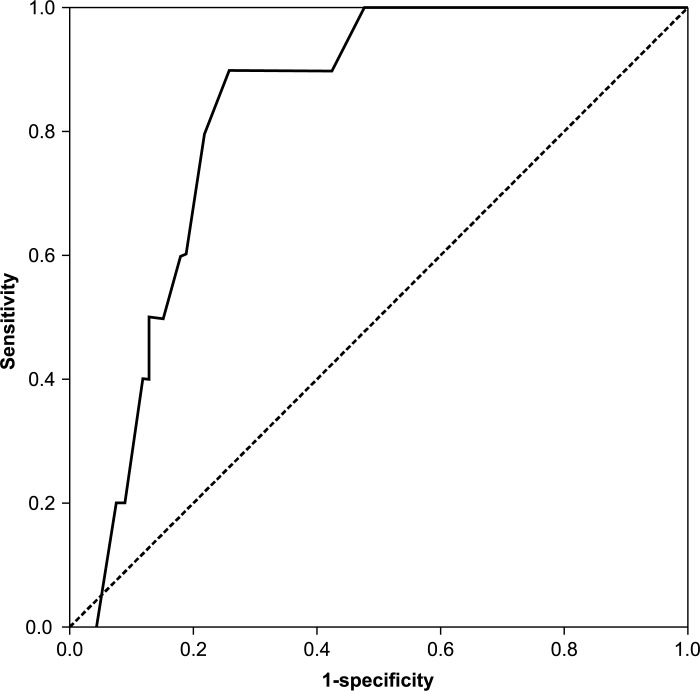
Receiver operating characteristic curve of age on the day of surgery.

**Fig 3:**
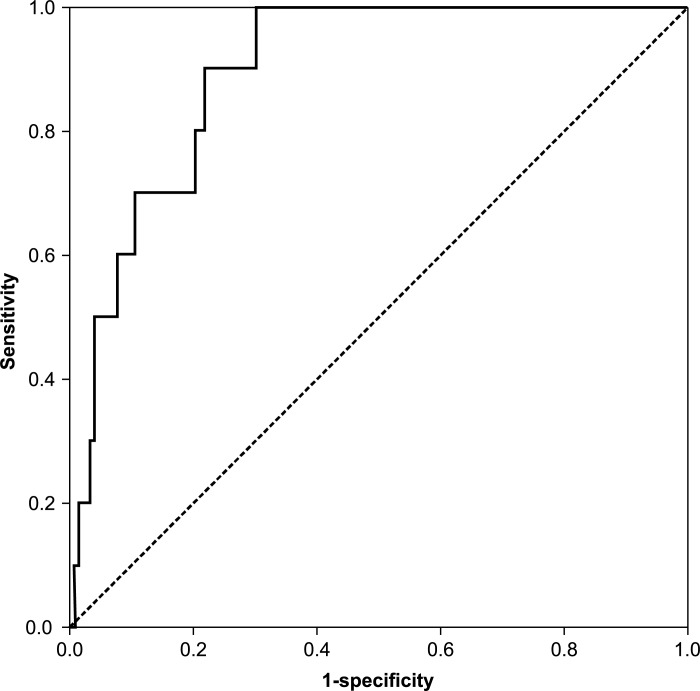
Receiver operating characteristic curve of the deep vein thrombosis index.

ROC curves were created for age, D-dimer value, and the value that was calculated by the following formula: DVT index = 0.12 × age (years) + 0.45 × the D-dimer value (μg/mL) (Figs 1–3). For the screening of DVTs, a DVT index of 8.15 was the most suitable cut-off value, as determined by the Youden index [14,15]. This value yielded 100% sensitivity and 70.1% specificity (Table 4). The cut-off value for age was 63.5, which caused a sensitivity of 88.9% and a specificity of 74.1%; and that of the D-dimer value was 1.85, which yielded a sensitivity of 60.0% and a specificity of 82.4% (Table 4).

**Table 4:** Area under the curve and cut-off value.

Independent variable	Cut-off value	Sensitivity*	Specificity*	AUC	95% CI	*p* value
Age	63.5 years	0.889	0.741	0.828	0.749–0.907	<0.001
D-dimer	1.85 μg/mL	0.600	0.824	0.716	0.522–0.910	0.021
DVT index^✝^	8.15	1.000	0.701	0.905	0.836–0.975	<0.001

AUC, area under the curve; CI, confidence interval; DVT, deep vein thrombosis.

*, Value at the cut-off value.

^✝^, DVT index = 0.12 × age (years) + 0.45 × D-dimer value (μg/mL).

With regard to the AUC, the DVT index was 0.905, and the AUCs were 0.828 and 0.716 for age and the D-dimer value, respectively. Therefore, the DVT index was the most accurate diagnostic test among the three tests.

## Discussion

Detecting DVTs preoperatively is necessary because approximately 90% of symptomatic PTE cases originate from a DVT in the lower limb [16]. If patients who are undergoing THA have a DVT, the DVT may be enlarged and/or expanded, which may subsequently cause PTE, a generally critical postoperative complication following arthroplasty. Therefore, venography or ultrasonography of the lower extremities must be performed in all patients before THA despite the limitations of these diagnostic tools [17].

In this study, the DVT index was calculated using the previously described formula, which showed 100% sensitivity and 70.1% specificity with an AUC of 0.905. This index was calculated using two coefficients of significant risk factors determined with multiple logistic regression. However, only age and D-dimer value demonstrated sensitivities of 88.9% and 60.0%; specificities of 74.1% and 82.4%; and AUCs of 0.831 and 0.712, respectively.

In previous reports, age-adjusted cut-off values by Schouten et al. [13] for the D-dimer value (0.1 × age [years]) [μg/mL] substantially increase specificity without modifying the sensitivity in patients suspected for DVT; a high sensitivity (>97%) was reported, but the specificity was <60%. If this Schouten’s index applied to the participants of our study, high specificity was observed (0.899), while sensitivity was 0.077. Moreover, Signorelli et al. [18] identified the risk of DVT by using routine blood tests, such as erythrocyte sedimentation rate, antithrombin III, C-reactive protein, D-dimer, and N-terminal pro-brain natriuretic peptide, in 295 consecutive medical patients. They reported that the DVT risk stratification system is moderately prognostic (AUC: 0.838; 95% confidence interval: 0.771–0.904; *p* < 0.001), whereas its sensitivity and specificity were 100% and 20%, respectively. In the present study, the DVT index was easy to calculate using only age and preoperative D-dimer value, and the sensitivity was 100%. The DVT index of all patients with a DVT was >8.15; therefore, patients with a DVT will never be missed, even in the absence of any clinical signs or symptoms associated with it.

Our DVT index may have a great potential benefit because DVT screening was performed with high accuracy in many patients. For example, in the current study, 150 of 224 patients had a DVT index <8.15; thus, ultrasonography or venography was not required for DVT screening.

The current study has several limitations. First, only a few patients were enrolled. Second, D-dimer concentrations at our institution are often measured using latex agglutination turbidimetry; conversely, in North America and Europe, they are measured using enzyme-linked immunoassay (ELISA). Latex agglutination turbidimetry is not recommended over the ELISA method, which may lead to lower sensitivity and specificity in this study. Finally, we did not examine the plasma levels of blood natriuretic peptide (BNP), a useful marker to detect increased pulmonary pressure. This marker is considered crucial for identifying patients at high risk for venous thromboembolism. However, given that BNP measurement is covered by health insurance in Japan for heart or renal failure only, we could not routinely examine BNP. Therefore, BNP evaluation was not included in this study.

In conclusion, this study showed that the DVT index is the most accurate screening method for the presence of DVTs before THA. This index was also easy to calculate and may be clinically useful. Nevertheless, further investigations are required to verify our findings and ensure more precise diagnoses.
